# Targeting Tumor Cells with Nanoparticles for Enhanced Co-Drug Delivery in Cancer Treatment

**DOI:** 10.3390/pharmaceutics13091327

**Published:** 2021-08-25

**Authors:** Wen-Ying Huang, Chih-Ho Lai, Shin-Lei Peng, Che-Yu Hsu, Po-Hung Hsu, Pei-Yi Chu, Chun-Lung Feng, Yu-Hsin Lin

**Affiliations:** 1 Department of Applied Cosmetology, Hung-Kuang University, Taichung 433304, Taiwan; beca690420@sunrise.hk.edu.tw; 2 Molecular Infectious Disease Research Center, Department of Microbiology and Immunology, Chang Gung University and Chang Gung Memorial Hospital, Taoyuan 333323, Taiwan; chlai@mail.cgu.edu.tw; 3 Department of Biomedical Imaging and Radiological Science, China Medical University, Taichung 404, Taiwan; speng@mail.cmu.edu.tw; 4 Department of Pharmacy, National Yang Ming Chiao Tung University, Taipei 112304, Taiwan; claire5588tv@gmail.com (C.-Y.H.); ositachu3@gmail.com (P.-Y.C.); 5 Center for Advanced Molecular Imaging and Translation, Chang Gung Memorial Hospital, Taoyuan 333, Taiwan; bloodbull@gmail.com; 6 Division of Hepatogastroenterology, Department of Internal Medicine, China Medical University Hospital, Taichung 404332, Taiwan; fjld6604@yahoo.com.tw; 7 Center for Advanced Pharmaceutics and Drug Delivery Research, Department and Institute of Pharmacology, Institute of Biopharmaceutical Sciences, Faculty of Pharmacy, National Yang Ming Chiao Tung University, Taipei 11221, Taiwan; 8 Department of Medical Research, China Medical University Hospital, China Medical University, Taichung 404333, Taiwan

**Keywords:** gastric cancer, nanoparticle, fucoidan, d-alpha-tocopherylpoly (ethylene glycol) succinate, combination chemotherapy

## Abstract

Gastric cancer (GC) is a fatal malignant tumor, and effective therapies to attenuate its progression are lacking. Nanoparticle (NP)-based solutions may enable the design of novel treatments to eliminate GC. Refined, receptor-targetable NPs can selectively target cancer cells and improve the cellular uptake of drugs. To overcome the current limitations and enhance the therapeutic effects, epigallocatechin-3-gallate (EGCG) and low-concentration doxorubicin (DX) were encapsulated in fucoidan and d-alpha-tocopherylpoly (ethylene glycol) succinate-conjugated hyaluronic acid-based NPs for targeting P-selectin-and cluster of differentiation (CD)44-expressing gastric tumors. The EGCG/DX-loaded NPs bound to GC cells and released bioactive combination drugs, demonstrating better anti-cancer effects than the EGCG/DX combination solution. In vivo assays in an orthotopic gastric tumor mouse model showed that the EGCG/DX-loaded NPs significantly increased the activity of gastric tumors without inducing organ injury. Overall, our EGCG/DX-NP system exerted a beneficial effect on GC treatment and may facilitate the development of nanomedicine-based combination chemotherapy against GC in the future.

## 1. Introduction

The stomach is located in the upper abdomen between the esophagus and small intestine. Its primary function is to store and partially digest food after ingestion [1,2]. Abnormal growth and multiplication of gastric cells can lead to gastric cancer (GC). Most GCs are adenocarcinomas that arise from the mucosal tissue lining inside the stomach. Abnormal cells may penetrate deeper into the stomach wall or spread to nearby organs or tissues [3]. Cell adhesion molecules are the major elements of the metastasis process [4]. In particular, P-selectin is an adhesion molecule that mediates the interaction of platelets and endothelial cells with monocytes, neutrophils, and tumor cells [5]. P-selectin also binds to several human cancers, such as GC, colon cancer, and breast cancer [6,7]. Cluster of differentiation (CD)44-positive GC cells show self-renewal properties and the ability to produce differentiated offspring, consistent with the cancer stem cell phenotype [8]. CD44-positive GC cells are reported to contribute to increased resistance to radiation- or chemotherapy-induced cancer cell death [9].

Cancer nanotechnology is being vigorously developed for applications in cancer imaging, molecular diagnosis, and targeted therapy [10,11,12]. Compared with their single- or dual-ligand-targeting nanoparticle (NP) counterparts, non-targeted NPs significantly increase metastasis deposition. Multiligand NPs are obtained by modulating the surfaces of NPs with more than one type of targeting ligand. These facilitate high-precision targeting of different metastatic subpopulations that express different targetable receptors otherwise missed by single-ligand NPs [13,14]. Fucoidan (FD) is the active ingredient of seaweed that induces cell apoptosis, inhibits the proliferation of cancer cells, and is reported to have a strong affinity for P-selectin, which interacts with sulfated oligosaccharides [15,16,17]. Hyaluronic acid (HA) is a natural linear polysaccharide, composed of repeating disaccharide units of β(1,3)-*N*-acetylglucosamine (GlcNAc) and β(1,4)-glucuronic acid (GlcUA), which forms van der Waals bonds and hydrogen bonds with migrating cells via the CD44 ligand receptors [18,19]. d-alpha-tocopheryl poly(ethylene glycol) (PEG) succinate (TPGS) is a water-soluble derivative of natural vitamin E and prevents P-glycoprotein (P-gp) adenosine triphosphatase (ATPase) from hydrolyzing ATP by blocking the ATP-binding sites, thus inhibiting ATPase activity [20]. Herein, we prepared a TPGS-conjugated HA (TH) copolymer that can simultaneously interact with membrane-bound CD44 and reduce P-gp expression in GC cells.

**Figure 1:**
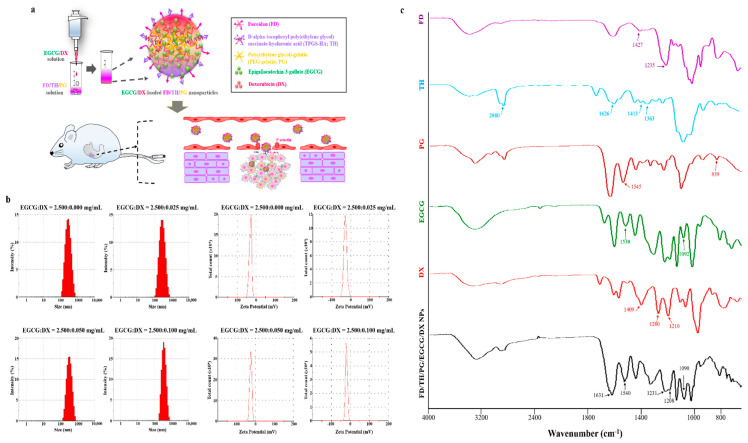
(**a**) NPs were prepared by adding EGCG/DX solution into FD/TH/PG solution under gentle stirring at 37 °C; the schematic representation of the strategy for using EGCG/DX-loaded FD/TH/PG NPs on carcinoma cells is shown; (**b**) Particle size distributions and zeta potential values of EGCG/DX-loaded FD/TH/PG NPs made with different DX concentrations; (**c**) Fourier transform infrared analyses of FD, TH, PG, EGCG, DX, and FD/TH/PG/EGCG/DX NPs.

Doxorubicin (DX), an anthracycline antibiotic, is currently the most effective chemotherapeutic drug used to treat GC, ovarian cancer, and breast cancer. DX acts as a DNA intercalator and topoisomerase II inhibitor. However, it exhibits strong cardiotoxicity [19,21]. The standard dose of 20 mg/m^2^ is widely used for GC, but an overall response to DX monotherapy is observed in only 17% of GC patients [22,23,24]. Epigallocatechin-3-gallate (EGCG), a bioactive polyphenol compound in green tea, has antioxidant and anti-inflammatory properties and can be utilized in disease management [25]. EGCG can sensitize the efficacy of DX, enhancing its therapeutic effect in hepatocellular or prostate cancer [26,27]. Combination chemotherapy is a promising method for improving cancer treatment. However, clinical combination therapy is limited by the unique pharmacokinetics of the combined drugs, resulting in uneven drug distribution [28,29]. We established tumor-targeting NPs, combining FD binding to P-selectin-expressing tumors and TH targeting CD44-expressing tumors to reduce P-gp expression. Gelatin is a natural polymer containing residues of glycine, proline, hydroxyproline, and alanine, which are common amino acids. Gelatin-polyphenol interactions primarily occur through hydrogen bonds between hydrophobic amino acids (mainly proline residues) and the phenol ring of polyphenols [30,31]. Thus, in this study, we used PEG-gelatin (PG) to load the anticancer agents EGCG and DX to enhance their loading efficiency and sustained release, thereby attempting to maximize their combined therapeutic effect and minimize systemic toxicity (Figure 1a).

## 2. Materials and Methods

### 2.1. Preparation and Characterization of EGCG/DX-Loaded FD/TH/PG NPs

TH and PG were synthesized according to the protocols suggested by Chen et al. [32]. For TH copolymer preparation, 0.2 mmol dicyclohexylcarbodiimide and 0.2 mmol 4-dimethylaminopyridine were dissolved in 5.0 mL of acetonitrile and added to an aqueous HA solution in deionized (DI) water (20.0 mg/mL in 10.0 mL). The solution was then stirred for 1.0 h to activate the carboxylic groups of HA. Subsequently, 0.1 g of TPGS was dissolved in 2.0 mL of DI water, then slowly added to the above mixed solution and stirred for 24.0 h under a nitrogen atmosphere. The PG copolymer was prepared by dissolving the 2.0 g of gelatin into 20.0 mL of dimethyl sulfoxide (DMSO). The resultant mixture was serviced as solvent for 0.6 g of methoxypolyethylene glycol succinimidyl ester (mPEG-NHS), and then stirred for 4 h. The unconjugated reagents were removed by dialyzing in 5.0 L of DI water, which was replaced five times per day. After the resultant TH and PG were lyophilized in a freeze dryer, Fourier transform infrared (FTIR) spectroscopy was used to detect the presence of purified compound in the sample.

NPs composed of the FD-TH complex with PG containing encapsulated EGCG and DX were prepared as follows. First, the EGCG-loaded FD/TH/PG NPs were formulated by optimizing the relative concentration of FD:TH. A series of FD:TH aqueous solutions (0.00:0.00, 1.20:1.20, 2.40:2.40 and 3.60:3.60 mg/mL in 0.50 mL) were added to aqueous PG solution (15.00 mg/mL in 0.50 mL) and then slowly shaken for 0.5 h at 37 ℃. Next, EGCG aqueous solution (5.00 mg/mL in 1.00 mL) was added to 1.0 mL of aqueous FD/TH/PG solutions. The reaction mixture was stirred for 30 min. Then, NPs with different FD:TH:PG:EGCG compositions (0.00:0.00:3.75:2.50, 0.30:0.30:3.75:2.50, 0.60:0.60:3.75:2.50, and 0.90:0.90:3.75:2.50 mg/mL) were completed. After encapsulating DX in these NPs, DX was dissolved in DI water (0.00, 0.10, 0.20, and 0.40 mg/mL in 0.50 mL) and then poured into the EGCG aqueous solution (10.00 mg/mL in 0.50 mL) to form EGCG/DX aqueous solutions (5.00:0.00, 5.00:0.05, 5.00:0.10, and 5.00:0.20 mg/mL in 1.00 mL). The mixture of EGCG/DX solutions (1.00 mL) and FD/TH/PG solution in DI water (1.00 mL) was stirred at 37 ℃ for 30 min. When the centrifugation was completed, the particle size distributions and zeta potentials of the obtained NPs were analyzed by a Zetasizer Nano apparatus (Malvern Instruments Ltd., Worcestershire, England, UK). The residual EGCG or DX content in the supernatant was assessed. The drug-loading efficiency of the NPs was determined using a reversed-phase high-performance liquid chromatography (HPLC) equipped with a C18 column and UV detector (230 nm).

### 2.2. Assessing pH Sensitivity and Evaluating Drug Release Profiles in EGCG/DX-Loaded FD/TH/PG NPs

The size distributions and morphological changes of NPs at pH 7.4, 6.5, and 5.0 were assessed using a Zetasizer instrument and transmission electron microscopy (TEM, JEOL Ltd., Tokyo, Japan) to evaluate the stability of the NPs. Buffers with pH 7.4, 6.5, and 5.0 were used to simulate the circulatory system, tumor tissue, and endosomal compartment environment, respectively [33]. The pH sensitivity of NPs was determined by using an HPLC system to measure the release of EGCG and DX under different pH environments. Briefly, 2.0 mg/mL of NP solution was immersed in a dialysis bag and dialyzed against different pH values of 5.0 (10.0 mM acetic acid/sodium acetate), 6.5, and 7.4 (10.0 mM phosphate-buffered saline (PBS)), respectively. The released solution was sampled at specific time intervals and replaced with an equal volume of fresh buffer. The amount of drug release under different pH environments was evaluated with a standard calibration curve and the experiment was repeated five times under each condition.

### 2.3. GC Cell Culture and the Anticancer Function

Stable luciferase-expressing human GC cells (Luc MKN45) were supplied by the Japanese Collection of Research Bioresources Cell Bank (JCRB No. JCRB1379). Luc MKN45 cells can be easily detected in animals using an in vivo imaging system (IVIS, PerkinElmer lnc., Waltham, MA, USA). The culture was started in a Petri dish at a seeding density of 3 × 10^5^ viable cells/mL in Roswell Park Memorial Institute (RPMI) 1640 medium supplemented with 10% fetal bovine serum (FBS) and 4 μg/mL of puromycin to maintain luciferase expression in a humidified atmosphere of 5% CO_2_/95% air at 37 ℃. The cells were harvested for subculture every three days with 0.25% trypsin plus 0.05% ethylenediaminetetraacetic acid (EDTA) solution and used for the cytotoxicity experiments. In the cell cytotoxicity experiment, Luc MKN45 cells were seeded in 96-well plates at a cell density of 1.0 × 10^4^ cells/well. After overnight incubation, the growth medium was replaced by PBS with 5% FBS containing distinct concentrations of EGCG solution (0–450 mg/L), DX solution (0–9 mg/L), EGCG/DX solution, or EGCG/DX-loaded NPs (EGCG concentration 0–450 mg/L; DX concentration 0–9 mg/L) for 2.0 h. The examined samples were extracted, and the cells were washed with PBS twice. After culturing in the growth medium for another 22.0 h, cells were incubated in growth medium with 1 mg/mL of 3-(4,5-dimethylthiazol-2-yl)-2,5-diphenyltetrazolium bromide (MTT) to evaluate the cell viability. The additive effect of the EGCG/DX combination on cancer cell viability was determined according to the Chou–Talalay method. The combination index (CI) was calculated using the following equation:

CI = D_E_/D_xE_ + D_D_/D_xD_, (1)

where D_E_ and D_D_ are the concentrations of EGCG and DX, respectively, used to achieve x% drug effect in the combined therapy. D_xE_ and D_xD_ are the concentrations of EGCG and DX, respectively, used alone to achieve x% drug effect. A CI value lower than 1.0 indicates a synergistic effect, a value equal to 1.0, an additive effect, and a value higher than 1.0, an antagonistic effect of the combined drug action [34,35,36]. The association between drug concentration and inhibitory concentration (IC) for cell viability reduction was calculated and an isobologram and a combination index (CI)—fraction affected (Fa) plot were created.

### 2.4. In Vitro Drug Cellular Uptake and Cell Distribution of NPs

Luc MKN45 cells carrying fluorescent-dye-conjugated EGCG and DX in NPs were assessed by flow cytometry for the quantification analysis of cellular uptake ratio of EGCG and DX. The cellular uptakes of DX and EGCG were determined by detecting the fluorescence of DX itself (EX470/EM585) and cyanine5 hydrazide (Cy5)-conjugated EGCG (Cy5-EGCG), respectively. The synthesis of fluorescent Cy5-EGCG was prepared by pouring Cy5 solution (1.0 mg/0.1 mL in DMSO) into EGCG aqueous solution (0.2 g/20.0 mL). After continuous stirring for 12.0 h, the fluorescence sample was freeze-dried. The 20.0 mL of DI water was added to the lyophilized Cy5-EGCG sample to precipitate and remove unreacted fluorescent dye. The suspension was centrifuged for several times for separating the precipitated Cy5 dye until no more fluorescent dye precipitation. The prepared Cy5-EGCG solutions were lyophilized with a freeze dryer. Fluorescent Cy5-EGCG/DX-loaded FD/TH/PG NPs were prepared as described before. In brief, Luc MKN45 cells were treated with Cy5-EGCG/DX solution and Cy5-EGCG/DX-loaded NPs for 2.0 h. The medium was washed with PBS and suspended and collected in 0.5 mL of PBS. A Becton Dickinson FACSCalibur flow cytometer (Becton Dickinson, Franklin Lakes, NJ, USA) was used to assess the Cy5-EGCG and DX content in 1.0 × 10^4^ cells, and the Cell Quest software WinMDI (Verity Software House, Inc., Topsham, ME, USA) was used for analysis.

The cellular distribution of fluorescent NPs was further observed by confocal laser scanning microscopy (CLSM, Zeiss LSM 880, Jena, Germany). Fluorescent rhodamine 6G-conjugated EGCG (Rh6G-EGCG) was prepared by the procedure of Cy5-EGCG. In addition, fluorescent polymer Cy5 hydrazide-labeled FD (Cy5-FD), Alexa594 hydrazide-labeled TH (Alexa594-TH), and Atto425 *N*-hydroxysuccinimide ester-labeled PG (Atto425-PG) were synthesized. These fluorescent solutions (Cy5, Alexa594, and Atto425; 1.0 mg/0.1 mL in DMSO) were gently poured into aqueous FD solutions, TH and PG (0.2 g/20.0 mL) in DI water, respectively. The reaction mixtures were stirred for 12.0 h and dialyzed against DI water in the dark for detaching the unconjugated fluorescent dye. The residue of Cy5-FD, Alexa594-TH, and Atto425-PG solutions were lyophilized in a freeze dryer to prepare distinct fluorescence samples. In brief, Luc MKN45 cells at a density of 3 × 10^5^ cells/cm^2^ were seeded on glass coverslips. After preculture for 24.0 h and incubation of fluorescent Rh6G-EGCG/DX solutions and Rh6G-EGCG/DX-loaded Cy5-FD/Alexa594-TH/Atto425-PG NPs for 2.0 h, the medium was washed with PBS. The cells were fixed with 3.7% paraformaldehyde (PFA), and the nuclei were stained with 4,6-diamidino-2-phenylindole (DAPI) and observed by CLSM.

### 2.5. Evaluation of NP Binding to P-Selectin and CD44s

To determine the capability of FD and TH in NPs in terms of the binding specificity of P-selectin and CD44, human recombinant P-selectin or CD44 (0.5 µg/50 µL) was added to high-hydrophobicity 96-well ELISA plates and incubated overnight at 4 °C. After being washed with PBS, the wells were blocked with 0.1 mL bovine serum albumin (BSA) for 1.0 h. After washing with PBS again, the wells were incubated with different concentrations of fluorescent Rh6G-FD/fluoresceinamine (FA)-TH/PG solution or Rh6G-FD/FA-TH/PG/EGCG NPs for 30 min and gently washed thrice with PBS. Fluorescence images were taken with an EnSpire™ (PerkinElmer lnc., Waltham, MA, USA) Multilabel Plate Reader by averaging 16 reads per well (excitation 525 nm/emission 548 nm for Rh6G-FD; excitation 480 nm/emission 520 nm for FA–TH). Test fluorescent sample solutions (400 µg/mL) and anti-P-selectin or anti-CD44 antibody (2.0 µg/mL) were added to the wells of the previously coated P-selectin or CD44 for the comparisons of binding specificity. The total volume of the resultant mixture in each well was 50 µL. After incubating for 0.5 h, fluorescence measurements were executed. The effect of the prepared NPs on cell surface protein expression was evaluated by culturing 3 mL of Luc MKN45 cells at a density of 3 × 10^5^/mL on glass coverslips for 24.0 h at 37 ℃.

The fluorescent Rh6G-FD/FA-TH/PG/EGCG NPs were treated to the cells for 2.0 h. Then, NPs were fixed in 1.0% PFA and stained for P-selectin or CD44 for immunofluorescence experiments. The fixed cells were blocked in PBS with 1.0% BSA and Tween 20 for 1 h, and a mixture solution of primary antibodies (rabbit anti-P-selectin antibody or mouse anti-CD44 antibody) was used for treatment overnight at 4 ℃. After washing with PBS, the incubation of cells in a mixture of anti-rabbit Cy5^®^ secondary antibody or anti-mouse Alexa Fluor^®^ 594 secondary antibody was performed for 1.0 h in the dark. Subsequently, the cells were uniformly mounted on slides and examined using CLSM.

### 2.6. Flow Cytometric Analysis of Cell Cycle and Western Blotting Investigation of Apoptosis-Related Proteins

The incubation of Luc MKN45 cells in the tested EGCG/DX-loaded NPs (EGCG concentration 0–200 mg/L; DX concentration 0–4 mg/L) for 2.0 h was used to evaluate the efficiency of EGCG/DX-loaded NPs on the cell cycle. Then, the cells were washed with PBS and incubated in cell cultured medium for 22.0 h. The cells were again washed with ice-cold PBS and fixed in ice-cold 70% ethanol. After the cells were washed with PBS for the third time, hypotonic buffer was utilized for suspending cells. Subsequently, cells were incubated for 1.0 h at 37 °C. The propidium iodide (PI; 1.00 mg/mL, 0.01 mL) was applied to the buffer, and the cells were again incubated for 0.5 h at 4 °C in the dark. The stages of the cell cycle were evaluated by PI and a Becton Dickinson FACSCalibur flow cytometer. After treating Luc MKN45 cells with EGCG/DX-loaded NPs with different drug concentrations, Western blotting analysis was utilized to assess the expression of apoptotic proteins. Cell lysis was performed by using freeze-thaw cycles, and the cell lysates were centrifuged at 6000× *g* at 4 °C. Cell protein lysates were separated into the same amounts with electrophoresis using sodium dodecyl sulfate-polyacrylamide gel electrophoresis, and the isolated proteins were resettled to polyvinylidene difluoride (PVDF) membranes. The PVDF membranes were put in 5% (*w*/*v*) nonfat dry milk buffer for 1.0 h and then probed with the following primary antibodies at 4 °C overnight: rabbit polyclonal anti-caspase-9 and anti-poly(ADP-ribose) polymerase (PARP), mouse monoclonal anti-caspase-3, and anti-α-tubulin. Finally, the PVDF membranes were incubated with horseradish peroxidase-conjugated secondary antibodies for 1.0 h, and the immune complexes were evaluated using enhanced chemiluminescence.

### 2.7. Estimation of Anti-Tumor Activity of Bioluminescent Imaging and IVIS Spectrum Detection on Nanoparticles in Tumors

Animal use and care in this study complied with the 1996 revision of the Guide for the Care and Use of Laboratory Animals prepared by the Institute of Laboratory Animal Resources, National Research Council and published by the National Academy Press. Male six-week-old mice with severe combined immunodeficiency (SCID) and weighing 25 g, purchased from the National Laboratory Animal Center, were placed in the Laboratory Animal Center at Yang Ming Chiao Tung University, Taiwan. The animal care guidelines and all experimental protocols were approved by the Institutional Animal Care and Use Committee (IACUC 1070307). The mice were kept under standard conditions (lights on from 6 AM to 6 PM) and allowed to adapt to local conditions for one week. To establish an orthotopic gastric tumor mouse model, the Luc MKN45 cells were inoculated via the orthotopic implantation method. In brief, after sterilizing the shaved abdominal skin, an incision was made to expose the stomach and the injection of Luc MKN45 cells (1 × 10^7^/100 μL) mixed in matrix gel (Becton Dickinson, Franklin Lakes, NJ, USA) suspension into the gastric wall was performed sub-serosally. Once the stomach was returned to the peritoneal cavity, the abdominal wall and skin were sutured closed.

After the stable detection of an orthotopic gastric tumor was achieved, drug treatment was initialized. The tumor growth was monitored using IVIS spectrum detection by capturing bioluminescence signals from Luc MKN45 cells. The mice were divided into three groups randomly with six mice each, with the injection of FD/TH/PG solution (control group), 20.0 mg/kg of EGCG/0.4 mg/kg DX in EGCG/DX solution, or EGCG/DX-loaded FD/TH/PG NPs via tail vein, respectively. Luciferin was injected intraperitoneally into the mice and waited for 10 min for bioluminescence expression and images were taken by IVIS. The quantification of the bioluminescence signals was used to monitor the tumor growth. The animals were sacrificed after the final observation and organs were taken for histological examination by hematoxylin and eosin (H&E) or immunohistochemical (IHC) staining. The efficiency of EGCG/DX-loaded NPs on tumor status was assessed by IHC staining of M30 (apoptosis marker) and Ki-67 (proliferation marker). After dewaxing the paraffin, the tissue sections were immersed in a series of concentrations of ethanol and xylene for rehydration. The sections were stained with primary antibodies of M30 CytoDeath (Peviva, Bromma, Stockholm, Sweden) and Ki67 (Thermo Fisher Scientific, Grand Island, NY, USA) after blocking with BSA. Then, secondary peroxidase antibodies were used for the probe. Finally, the distribution expression of M30 and Ki67 and tissue inflammation at different magnifications were observed under a light microscope. Meanwhile, in the in vivo NP distribution study, fluorescent-labeled Rh6G-EGCG/DX-loaded Cy5-FD/Alexa594-TH/VioTag750-PG NPs were injected via tail vein to evaluate their distribution during the treatment. After euthanizing the mice at 24 h, fluorescence images of organs and tissues were obtained in the IVIS. Moreover, the mice also received fluorescent Rh6G-FD/FA-TH/PG/EGCG NPs via 24.0 h intravenous injection. Subsequently, the gastric tumor slides were assessed using immunofluorescence staining with rabbit anti-P-selectin antibody or mouse anti-CD44 primary antibodies, probed with secondary antibodies (anti-rabbit Cy5^®^ or anti-mouse Alexa Fluor^®^ 594), then examined using CLSM.

### 2.8. Statistical Analysis

One-way analysis of variance was used to analyze differences in the measured characteristics of the treatment groups. The confidence intervals were determined using Statistical Analysis System, version 6.08 (SAS Institute, Cary, NC, USA). The mean ± standard deviation (SD) was used to represent all data and *p* < 0.05 was considered statistically significant.

## 3. Results

### 3.1. Preparation and Characterization of EGCG/DX-Loaded FD/TH/PG NPs

To create NPs that can be combined with EGCG and DX, first, the optimal material FD:TH ratio should be determined when preparing EGCG-loaded NPs and then the concentration of DX should be adjusted to create EGCG/DX-loaded NPs. As shown in Table 1, as the amount of FD/TH incorporated in FD/TH/PG/EGCG NPs increased, the mean particle size of the NPs was 214.68 ± 18.78 to 741.79 ± 198.79 nm and their polydispersity index (PDI) was 0.25 ± 0.08 to 0.95 ± 0.26. When the FD:TH:PG ratio was 0.60:0.60:3.75 mg/mL, the NPs formed a dissoluble opalescent suspension after centrifugation. The particle size was 224.68 ± 18.78 nm and the zeta potential −26.14 ± 1.75 mV, with a significantly narrow distribution (0.25 ± 0.08) (Table 1). However, when the FD:TH:PG ratio increased to 0.90:0.90:3.75 mg/mL, the average particle size of the NPs increased, as did their PDI (to 0.4), indicating high heterogeneity. To further load DX into NPs, NPs containing different concentrations of EGCG and DX were prepared using a gelation technique in which the EGCG/DX solution was added to the FD/TH/PG aqueous solution. The HPLC analysis method showed that, at an EGCG:DX ratio of 2.50:0.05 mg/mL, the loading efficiency of EGCG and DX in the NPs was 59.07% ± 7.06% and 63.46% ± 4.59%, respectively. The subsequent experiment required a narrower distribution (PDI = 0.21 ± 0.05) (Table 2 and Figure 1b).

In FTIR investigates of EGCG/DX-loaded FD/TH/PG NPs, the representative peaks at 1235 and 1427 cm^−1^ ascribed to FD were dispensed to sulfate esters (O=S=O) and CH_2_ (galactose, xylose) scissoring vibrations or CH_3_ (fucose, O-acetyls) asymmetric bending vibrations. The synthesized TH copolymer was analyzed with FTIR and showed representative peaks at 1363 and 2880 cm^−1^, conforming to the −CH_2_ group on PEG and the –CH stretching band of TPGS, and peaks at 1413 and 1626 cm^−1^, reflecting the contribution of C–O stretches symmetrically and C=O stretches asymmetrically, corresponding to the carboxyl group on HA. In the PEG-conjugated gelatin (PG) spectrum, characteristic peaks at 839 and 1545 cm^−1^ were observed, corresponding to bending C–C and –NH stretching vibrations on PEG and gelatin. Finally, EGCG/DX-loaded FD/TH/PG complex spectra showed characteristic peaks at 1090 and 1208 cm^−1^, corresponding to the C–OH deformation of EGCG and DX, respectively. Moreover, the NH bending peak on PG shifted from 1545 cm^−1^ to 1540 cm^−1^; the S=O or C=O stretching peaks on FD or TH shifted from 1235 cm^−1^ or 1626 cm^−1^ to 1231 cm^−1^ or 1631 cm^−1^, respectively. This indicates that the H atoms of EGCG or DX interacted via hydrogen bonds with the N atoms of PG (C−OH···N−C) or the O atoms of FD or TH (C−OH···O=S or O=C) (Figure 1c).

### 3.2. Characterization of the Morphology and Drug Release Profile of EGCG/DX-Loaded FD/TH/PG NPs

**Figure 2:**
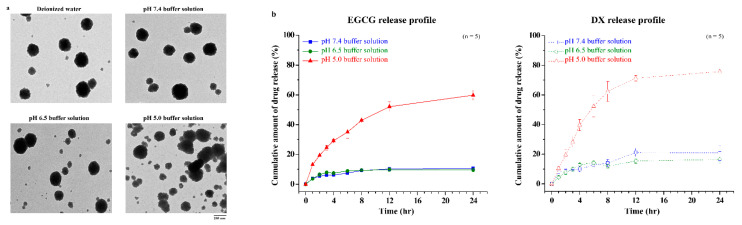
(**a**) Transmission electron micrographs of nanoparticles in buffer solution with different pH values; (**b**) The release profile of EGCG and DX in varying pH environments at 37 ℃. Data is expressed as mean ± SD (*n* = 5).

We evaluated the pH sensitivity of the NPs and the distinct drug release profile of EGCG/DX-loaded NPs using TEM and HPLC, respectively (Figure 2). At pH 7.4 and 6.5 (simulating the physiological fluids and tumor tissues), the NPs shown as stable spheres with a matrix structure with particle sizes of 200–230 nm, because EGCG and DX form hydrogen bonds with PG or TH. The drug release percentage from NPs over 3.0 h incubation was 6.21% ± 0.87% (EGCG) and 9.54% ± 0.15% (DX) at pH 7.4 and 7.94% ± 0.69% (EGCG) and 9.87% ± 1.35% (DX) at pH 6.5. At pH 5.0 (simulating an acidic environment in endosomal of cancer cells), some of the –COO– groups were moderately protonated in TH, affecting the negative electrostatic repulsion of NPs, which become unstable and partially collapse but with increased particle size (418.31 ± 69.75 nm) (Figure 2a). Under those conditions, the EGCG release was 24.58% ± 1.64% and the DX release was 28.17% ± 3.68% within 3.0 h. In addition, the hydrogen bonding between the N or O atom in PG or TH, and the H atom in the hydroxyl group of EGCG or DX caused slower drug release from EGCG/DX-loaded NPs, reaching 59.74% ± 3.31% and 75.74% ± 2.08% for EGCG and DX, respectively, over 24.0 h (Figure 2b).

### 3.3. Effects of FD/TH Solutions and EGCG/DX-Loaded FD/TH/PG Nanoparticles on Binding of P-Selectin and CD44

**Figure 3:**
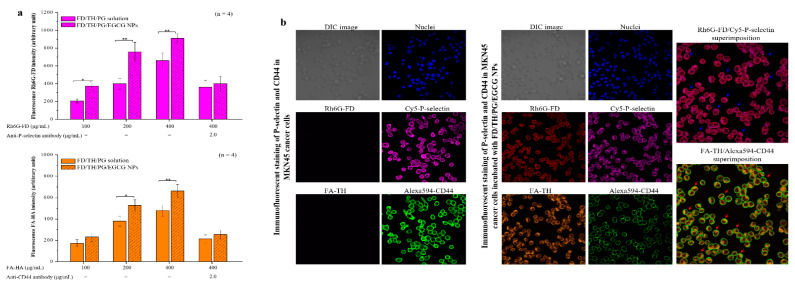
(**a**) Binding assays of fluorescence Rh6G-FD and FA-TH to immobilized recombinant P-selectin and CD44 protein. Data is expressed as mean ± SD (*n* = 4). Asterisk symbols * and ** represent statistically significant differences with *p* value < 0.05 and < 0.01, respectively; (**b**) Immunofluorescence staining of P-selectin and CD44 proteins in MKN45 cancer cells incubated with Rh6G-FD/FA-TH/PG/EGCG NPs.

To study the targeting of P-selectin and CD44 by Rh6G-FD/FA-TH/PG solution or Rh6G-FD/FA-TH/PG/EGCG NPs with different FD or TH molecule concentrations (100, 200 and 400 μg/mL), their fluorescence intensity was quantified by spectrofluorometry. The evident fluorescence intensities at Rh6G-FD or FA-TH concentrations of 100 μg/mL to 400 μg/mL were 207.19 ± 24.09 to 659.21 ± 85.31 or 173.39 ± 35.19 to 480.11 ± 51.26 (only FD/TH/PG solutions), respectively and 373.91 ± 59.08 to 911.21 ± 52.61 or 231.39 ± 40.09 to 662.31 ± 60.76 (EGCG/DX-loaded FD/TH/PG NPs), respectively. Additionally, Rh6G-FD or FA-TH fluorescence analysis indicated that the contact degree of EGCG/DX-loaded NPs with P-selectin or CD44 was greater than that of FD/TH/PG solutions. The addition of P-selectin or CD44 reduced the adhesion of targeting proteins to FD or TH by approximately 50% due to blockage by the competing anti-targeting P-selectin or CD44 protein antibodies (Figure 3a). Therefore, we evaluated the benefits of the relative composition of FD and TH in P-selectin- and CD44-targeted NPs. The prepared NPs interact with protein expression on GC cell surface was observed by CLSM. After 2.0 h of incubation with fluorescent Rh6G-FD/FA-HA/PG/EGCG NPs, fluorescence signals (Rh6G-FD (red dot) and FA-TH (orange dot)) of the NPs were observed in the cell cytoplasm and intercellular spaces, and then the NPs could exhibit co-localize and interact with P-selectin (purple dots) and CD44 (green dots) in gastric cancer cells (blue or red arrows indicate superimposed red/purple or orange/green dots) (Figure 3b).

### 3.4. Evaluating the Anticancer Effect of EGCG/DX-Loaded FD/TH/PG NPs and the Combination Index Determination

**Figure 4:**
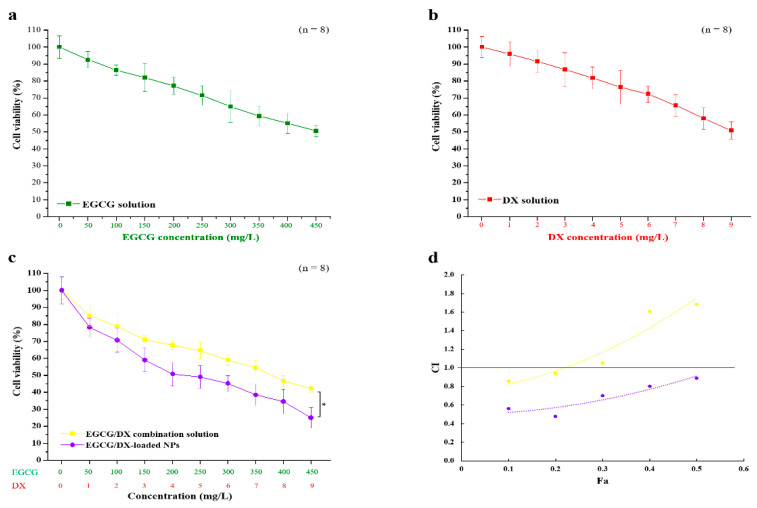
The effects of EGCG, DX, EGCG/DX combination solution, and EGCG/DX-loaded NPs on Luc MKN45 cell viability: (**a**) A 2.0 h dose response treatment with EGCG; (**b**) A 2.0 h dose response treatment with DX; (**c**) A 2.0 h dose response treatment with EGCG/DX solution and EGCG/DX-loaded NPs; cell viability was assessed at 24.0 h for a–c; data are expressed as mean ± SD (*n* = 8). Asterisk * represents statistically significant difference with *p* value < 0.05. (**d**) Combination index–fraction affected (CI-Fa) plot; CI < 1.0 indicates synergistic effects, CI = 1.0 indicates additive effects and CI > 1.0 indicates antagonistic effects of the combined drug action. Dots below the slanted line indicate synergistic effects.

To evaluate the potential inhibitory effect of EGCG or DX alone and the synergy of the EGCG/DX solution, the effects of EGCG/DX-loaded NP treatment on GC cell viability was analyzed. Both EGCG and DX significantly affected cell growth at high concentrations of 450 and 9 mg/L, respectively, and the cell viability decreased to 50.59% ± 3.46% and 50.81% ± 5.11%, respectively (Figure 4). We also compared the efficacy of EGCG/DX solution and EGCG/DX-loaded NP on inhibiting GC growth. The cell survival after EGCG/DX-loaded NP treatment was obviously lower than that after EGCG/DX solution treatment. The anti-cancer activity of EGCG/DX-loaded FD/TH/PG NPs (EGCG concentration, 150–450 mg/L; DX concentration, 3–9 mg/L) on Luc MKN45 cells was significantly higher than that of the EGCG/DX combined solution (*p* < 0.05; Figure 4c). The therapeutic IC50 of EGCG/DX-loaded NPs was 200/4 mg/L, which was half that of the EGCG/DX combined solution (400/8 mg/L).

In addition, the CI-Fa plot and isobologram are described in Figure 4d. EGCG/DX combination treatment had a slightly synergistic effect on the cell viability, with CI of IC_10_ and IC_20_ being 0.86 and 0.94, respectively. EGCG/DX-loaded NP treatment showed a better synergistic antiproliferation effect, with CIs of IC_10_, IC_20_ IC_30_, IC_40_, and IC_50_ being 0.56, 0.48, 0.70, 0.80, and 0.89, respectively, compared with EGCG/DX combination solution. This result showed that GCG/DX-loaded FD/TH/PG NP targeting cancer therapy has better benefits than EGCG/DX combined drug therapy.

### 3.5. Internalization of EGCG and DX and Cellular Uptake of Complexes Contained within Fluorescent Nanoparticles

**Figure 5:**
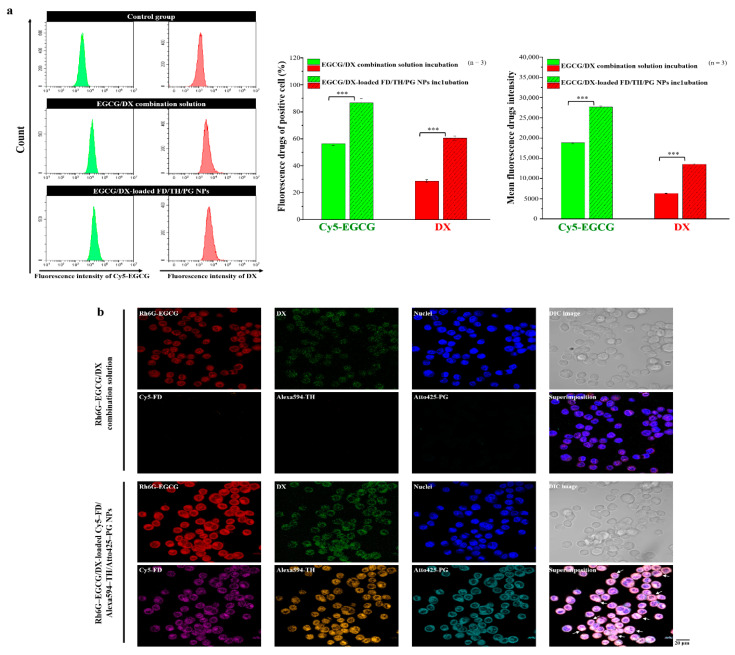
(**a**) In vitro cellular uptake of Cy5-GCG and DX was assessed using flow cytometry after treatment with EGCG/DX combination solution and EGCG/DX-loaded NPs; (**b**) Confocal images of gastric cancer cells viewing cellular distribution of the Rh6G-EGCG/DX combination solution and Rh6G-EGCG/DX-loaded Cy5-FD/Alexa594-TH/Atto425-PG NPs, *** represent statistically significant differences with *p* value < 0.001.

To confirm the cellular uptake ability, fluorescence-activated cell sorting analysis was made after the cells were treated with both the drug combination and NPs containing Cy5-EGCG and DX. Flow cytometry examination was performed with Luc MKN45 cells after treatment with FD/TH/PG NPs loaded with EGCG/DX. The cellular uptake and fluorescence intensity of Cy5-EGCG were 86.53% ± 3.25% and 27,639.98 ± 254.91, respectively, while those of DX reached 60.42% ± 1.56% and 13,493.23 ± 143.87, respectively, after exposure for 2.0 h. In contrast, after treatment with only EGCG/DX solution, the cellular uptake and total fluorescence intensity of Cy5-EGCG were 56.29% ± 1.32% and 18,783.54 ± 198.40, respectively and those of DX were 28.62% ± 0.97% and 6275.41 ± 60.43, respectively (Figure 5a). This difference in drug absorption capacity is closely related to the presence of EGCG/DX in NPs, which successfully identified the specific targets, namely P-selectin and CD44, on the cell surface.

CLSM was used to compare and evaluate the delivery efficiency of EGCG/DX solution and EGCG/DX-loaded FD/TH/PG NPs after 2.0 h treatment, and DAPI with fluorescence expression was used to stain cell nuclei. Cy5-FD, Alexa594-TH, and Atto425-PG were co-localized in the cells. The internalization of EGCG and DX was evaluated by observing Rh6G-EGCG and DX fluorescence performance signals. Drug content (Rh6G-EGCG and DX) was higher in GC cells treated with the EGCG/DX-loaded NPs than in those treated with the EGCG/DX solution, indicating that the NP system infiltrated cancer cells and improved the delivery of EGCG and DX (Figure 5b).

### 3.6. Cell Cycle Arrest and Apoptosis Effects of NPs Loaded with EGCG/DX

**Figure 6:**
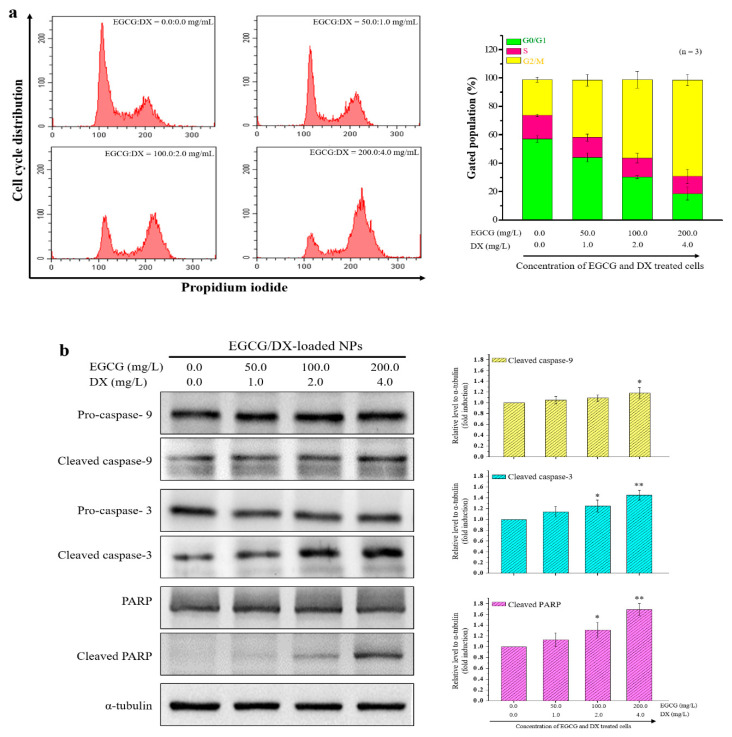
(**a**) The proportions of cells in the G0/G1, S, and G2/M phases, after treatment with EGCG/DX-loaded FD/TH/PG NPs are indicated. The cells were stained with propidium iodide and the cell cycle distribution was analyzed using flow cytometry. (**b**) Western blotting analysis of apoptosis-related caspase-9, caspase-3, and PARP in MKN45 cells after incubation with EGCG/DX-loaded FD/TH/PG NPs and α-tubulin was used as an internal control. * Statistically significant difference of *p* < 0.05 and ** *p* < 0.01, respectively as compared with the without sample treatment group.

To investigate whether the decrease in the growth rate of cells treated with test samples is associated with cell cycle arrest, we analyzed PI-stained Luc MKN45 cells treated with EGCG/DX-loaded NPs containing different EGCG and DX concentrations within 24.0 h by flow cytometry. As shown in Figure 6a, after EGCG/DX-loaded NP treatment, the cells displayed significant accumulation in the G2/M phase compared to the control group. In particular, EGCG/DX-loaded NPs significantly reduced the G0/G1 population (from 57.28% ± 2.35% to 18.66% ± 4.51%), the number of cells undergoing the S phase slightly decreased (from 16.48% ± 0.87% to 12.11% ± 5.03%), and the accumulation of G2/M in cells (from 25.11% ± 1.73% to 67.86% ± 3.95%) increased in an EGCG/DX dose-dependent manner, indicating that EGCG/DX-loaded NPs affect cell cycle arrest at the G2/M phase in GC cells. To determine whether the EGCG/DX-loaded NPs can induce cell apoptosis, we performed Western blotting and evaluated the ratios of cleaved caspase-9, caspase-3, and poly(ADP-ribose) polymerase (PARP) levels to their total protein expression (cleaved form plus proform). α-Tubulin was used as an internal control. Twenty-four hours after the samples were treated with different drug concentrations, EGCG/DX (200/4 mg/mL) was found to increase the cleaved protein to total protein expression ratios of caspase-9, caspase-3, and PARP by approximately 18%, 45%, and 69%, respectively, compared with that in the untreated group (Figure 6b).

### 3.7. Bioluminescent Imaging to Evaluate Anticancer Activity in an Orthotopic Gastric Tumor Mouse Model

**Figure 7:**
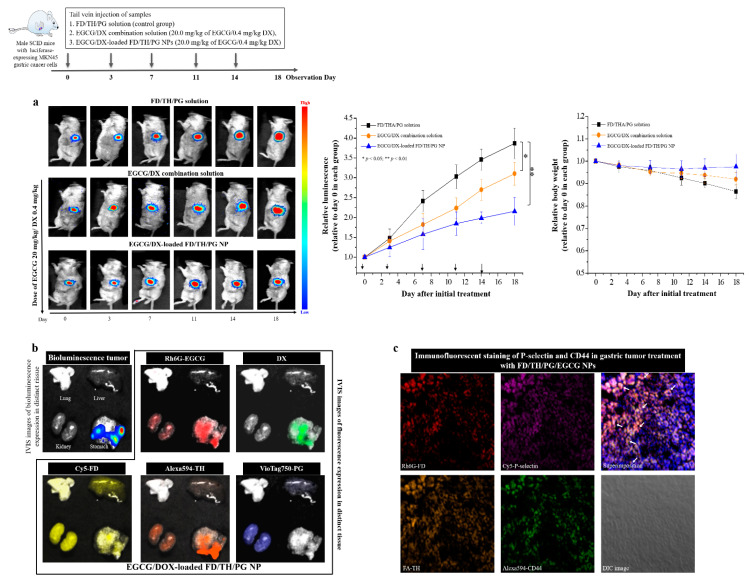
(**a**) The antitumor effects of different samples in orthotopic Luc MKN45 xenograft mice model. Mice were divided into three groups of six mice each and treated with FD/TH/PG solution (■), EGCG/DX combination solution (●), or EGCG/DX-loaded FD/TH/PG NPs (▲). * Statistically significant difference of *p* < 0.05 and ** *p* < 0.01. (**b**) Distributions of fluorescent Rh6G-EGCG/DX-loaded Cy5-FD/Alexa594–TH/VivoTag750-PG in tumor and organs after treatment. Gastric tumors were detected with a bioluminescent in vivo imaging system. (**c**) Immunofluorescence staining of P-selectin and CD44 proteins in gastric tumor slides treated with Rh6G–FD/FA-TH/PG/EGCG NPs.

We examined tumor-specific effects of EGCG/DX-loaded NPs in the gastric tumor mouse model of a luciferase-expressing gastric tumor. Male SCID mice were inoculated with EGCG/DX combination solution (20 mg/kg EGCG/0.4 mg/kg DX) and EGCG-loaded FD/TH/PG NPs or FD/TH/PG solution as a control by using intravenous tail injection every three days for a total of five injections. The bioluminescence signals of gastric tumor pointedly increased by 3.87-fold ± 0.39-fold (FD/THA/PG solution) and 3.10-fold ± 0.29-fold (EGCG/DX solution) at day 18. Furthermore, EGCG/DX-loaded FD/THA/PG NPs (2.16 ± 0.35 units) better inhibited tumor growth, with a lower relative photon flux compared with mice in other treatment groups (Figure 7a). There was no difference in body weight loss in our EGCG/DX-loaded NPs group and it was also superior to that in the FD/TH/PG control solution, indicating significant antitumor activity (Figure 7a). We also used IVIS to further evaluate the local accumulation of NPs. After fluorescent Rh6G-EGCG/DX-loaded Cy5–FD/Alexa594-TH/VioTag750-PG NPs were used for tail vein injection, fluorescent signals were detected on different tissues by IVIS technology. The gastric tumor was visualized by detecting bioluminescent signals formed by stable luciferase-expressing Luc MKN45 cells. The intensity of the fluorescent signals (Rh6G-EGCG and DX) at the tumor site significantly increased locally within 24.0 h, indicating continuous and sustained drug delivery by NPs targeting P-selectin and CD44 in the tumor. After 24.0 h, the mice were sacrificed to determine the total fluorescent counts in tumor and normal tissues and an increase in the NP distribution in the tumor led to a decrease in their distribution in other organs. Therefore, the tumor-targeting ability of EGCG/DX-loaded FD/TH/PG NPs was effective in the orthotopic gastric tumor mouse model (Figure 7b).

### 3.8. Histological and Immunohistochemical Analyses of Cancer Biomarkers

**Figure 8:**
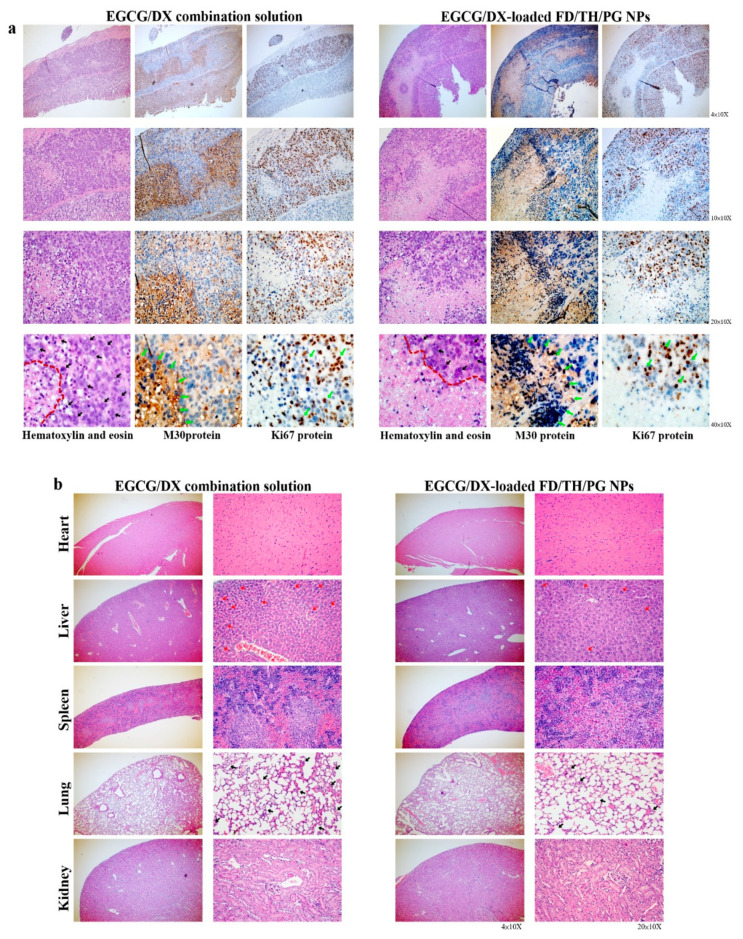
(**a**) Histological analysis and immunohistochemical analysis of Ki67 (proliferation marker) or M30 (apoptosis marker) of gastric tumor biopsy after treatment with EGCG/DX combination solution or EGCG/DX-loaded FD/TH/PG NPs; (**b**) Histological analysis of different organs in mice bearing gastric tumor after treatment with EGCG/DX combination solution or EGCG/DX-loaded FD/TH/PG NPs.

After the experimental mice were sacrificed, H&E staining was used for histological examination of gastric tumors and other organ biopsies (Figure 8). Compared with the EGCG/DX-loaded NP groups, tumor sections from the EGCG/DX combination group had more granular eosinophil infiltration (black arrow). The EGCG/DX-loaded NP group showed increased tumor necrosis, characterized as grade 2 tissue necrosis (necrosis of tumor cells or disappearance of more than 2/3 cells; the right side of the red line) compared with the EGCG/DX combination group (Figure 8a). Additionally, immunohistochemical analysis presented that after EGCG/DX-loaded FD/TH/PG NP treatment, M30 expression in tumors increased, while Ki67 expression diminished (i.e., coffee dots; green arrows, Figure 8a). These findings showed that targeted NPs induce GC cells apoptosis, resulting in a significant increase in anticancer activity with tumor necrosis. In the EGCG/DX combination group, the lung tissue showed inflammatory cell exudation, red blood cells scattered in multiple alveolar cavities, and thickened interstitial pulmonary edema (i.e., black arrows, Figure 8b). In contrast, the pathological damage was evidently minor in the EGCG/DX-loaded NP group, in which the alveolar structures were clear, and signs of inflammation decreased (i.e., black arrows, Figure 8b). Moreover, the hepatocyte swelling density and neutrophil infiltration situation of liver tissue biopsies in the EGCG/DX-loaded NP group was less than those in the EGCG/DX combined group (i.e., red arrows, Figure 8b). Therefore, targeting NPs can not only significantly improve the antitumor ability of drugs against GC, but also reduce the inflammatory response in the body.

## 4. Discussion

Cell adhesion is the process by which cells interact and attach to neighboring cells through specialized molecules, regulating cell motility and migration [37,38]. Adhesion molecules of the selectin family (E-, L-, and P-selectin) are a class of membrane-bound protein that participate in the initial adhesion of leukocytes to the site of infection or inflammation [37]. P-selectin expression increases in the vasculature of human cancers and facilitates metastasis by promoting the adhesion of circulating cancer cells to activated platelets and endothelial cells in distant organs [39,40]. P-selectin can also bind with high affinity to some sulfatides and sulfated polysaccharides, such as heparin, dextran sulfate, and FD [41,42]. Park et al. used FD extracted from *Fucus vesiculosus* to cause autophagic cell death of human GC cells and show antiproliferation activity via Beclin1 upregulation and the conversion of microtubule-associated protein (MAP) light chain 3-I (LC3-I) to LC3-II [43]. P-selectin binds to CD44 and promotes the capture of vascular endothelial cells and cancer cells by leukocytes [44]. HA, a major extracellular matrix component, is a physiological ligand that is overexpressed in CD44 on the surface of malignant tumor cells and can be used to selectively activate multiple oncogenic signaling pathways that lead to tumor cell-specific phenotypes [45]. CLSM showed that the fluorescence signals (Rh6G-FD (red dot) and FA-TH (orange dot)) of the prepared NPs were co-localized and interacted with P-selectin (purple dot) and CD44 (green dot) in gastric tumor tissue (white arrows)(Figure 7c). Rh6G-EGCG and DX fluorescence signals emanated by Rh6G-EGCG/DX-loaded Cy5-FD/Alexa594-TH/Atto425-PG NPs were attached to Luc MKN45 cells, and more drugs (EGCG and DX) were released into the cell cytoplasm compared with EGCG/DX combination treatment (Figure 5). Furthermore, the carrier EGCG/DX induced cell cycle arrest in the G2/M phase, inhibited GC proliferation, and enhanced the expression of apoptosis-related proteins (Figure 6).

Tumor cell growth generates a complex physiological environment that is distinct from regions surrounding healthy tissues. Both the extracellular and intracellular pH values of tumor cells are considered to be acidic and lower than those of normal cells [46,47]. Physiological pH is generally believed to be in the range of 7.2–7.4 for normal tissues and the circulatory system [48]. However, the pH value of tumor cells is weakly acidic, between 6.5 and 7.0, and the pH value of endosomes and lysosomes is between 5.0 and 6.0 [49]. Therefore, the pH of the tumor microenvironment and tumor cells plays an important role in the development and treatment of cancer. The NP system adopts a pH-sensitive adjustment strategy to prevent the untimely release of drugs. In the present study, NPs containing EGCG/DX were designed with a matrix structure that minimizes drug release into normal physiological environments, such as nontarget tissues and blood, and promotes the release of EGCG and DX into lysosomes and late endosomes of the tumor cells. The cumulative release of EGCG and DX at pH 5.0 amounted to 59.74% ± 3.31% and 75.74% ± 2.08%, respectively (Figure 2). Specifically, in vivo experiments confirmed that the lower bioluminescence intensity after intravenous injection of EGCG/DX-loaded NP (20 mg/kg EGCG/0.4 mg/kg DX) treatment significantly inhibited tumor growth (Figure 7). Histological analyses showed that the delivery of EGCG/DX to tumors through NPs can induce GC cell apoptosis, as indicated by increased levels of caspase-cleaved cytokeratin 18 (M30, ccK18) as an apoptotic cell death pharmacodynamic biomarker, accompanied by a reduction in inflammatory pulmonary lesions (Figure 8).

## 5. Conclusions

In summary, we applied an FD-based and TH-based NP system for the active, targeted co-delivery of EGCG and DX through P-selectin and CD44 ligand recognition to GC cells. Drug release was regulated by the NP system using a pH-sensitive adjustment strategy. In addition, the NPs prepared for the co-drug delivery system significantly improved the synergistic anti-cancer effect of EGCG and DX compared with that of the EGCG/DX combination solution. Targeting enhanced NP distribution in GC cells and decreasing it in major organs in the orthotopic gastric tumor could lead to the development of novel cancer therapies and facilitate their clinical trials.

## Figures and Tables

**Table 1:** Effect of different FD/TH proportions on particle sizes, polydispersity indices, zeta potential values, and drug loading efficiency of the prepared EGCG-loaded FD/TH/PG NPs (*n* = 5). ■ Precipitation of aggregates after centrifugation was observed.

PG at 3.75 mg/mL; EGCG at 2.50 mg/mL				
FD:TH (mg/mL)	Mean Particle Size (nm)	Polydispersity Indices	Zeta Potential Value (mV)	Loading Efficiency (%)
0.00:0.00	■	■	■	■
0.30:0.30	741.79 ± 198.79	0.95 ± 0.26	−17.72 ± 5.67	59.86 ± 9.87
0.60:0.60	214.68 ± 18.78	0.25 ± 0.08	−26.14 ± 1.75	63.48 ± 7.39
0.90:0.90	315.27 ± 47.84	0.41 ± 0.13	−29.76 ± 4.65	69.87 ± 3.14

**Table 2:** Effect of varying DX concentration on EGCG/DX-loaded FD/THA/PG particle sizes, polydispersity indices, zeta potential values, and drug loading efficiency (*n* = 5).

FD at 0.60 mg/mL; TH at 0.60 mg/mL; PG at 3.75 mg/mL					
EGCG:DX (mg/mL)	Mean Particle Size (nm)	Polydispersity Indices	Zeta Potential Value (mV)	Loading Efficiency (%)	
				EGCG	DX
2.500:0.000	214.68 ± 18.78	0.25 ± 0.08	−26.14 ± 1.75	63.48 ± 7.39	ND
2.500:0.025	205.43 ± 11.95	0.28 ± 0.06	−25.84 ±3.23	61.74 ± 3.49	59.43 ± 9.34
2.500:0.050	221.26 ± 13.26	0.21 ± 0.05	−23.30 ± 2.13	59.07 ± 7.06	63.46 ± 4.59
2.500:0.100	254.84 ± 18.75	0.38 ± 0.10	−22.87 ± 3.58	57.39 ± 8.74	67.78 ± 9.62
